# *ClbG* in Avian Pathogenic *Escherichia coli* Contributes to Meningitis Development in a Mouse Model

**DOI:** 10.3390/toxins13080546

**Published:** 2021-08-06

**Authors:** Peili Wang, Jiaxiang Zhang, Yanfei Chen, Haoran Zhong, Heng Wang, Jianji Li, Guoqiang Zhu, Pengpeng Xia, Luying Cui, Jun Li, Junsheng Dong, Qingqing Gao, Xia Meng

**Affiliations:** 1 College of Veterinary Medicine, Yangzhou University, Yangzhou 225009, China; wangpeilino1@sina.com (P.W.); jiaxiangzh_ang@sina.cn (J.Z.); chenmanbuzhidao@sina.cn (Y.C.); zhongaoran123@sina.cn (H.Z.); wh@yzu.edu.cn (H.W.); jjli@yzu.edu.cn (J.L.); yzgqzhu@yzu.edu.cn (G.Z.); ppxia@yzu.edu.cn (P.X.); lycui@yzu.edu.cn (L.C.); ydjunli@sina.cn (J.L.); 007601@yzu.edu.cn (J.D.); qqgao@yzu.edu.cn (Q.G.); 2 Jiangsu Co-Innovation Center for Prevention and Control of Important Animal Infectious Diseases and Zoonoses, Yangzhou 225009, China

**Keywords:** colibactin, *clbG*, *Escherichia coli* meningitis, APEC

## Abstract

Colibactin is a complex secondary metabolite that leads to genotoxicity that interferes with the eukaryotic cell cycle. It plays an important role in many diseases, including neonatal mouse sepsis and meningitis. Avian pathogenic *Escherichia coli* (APEC) is responsible for several diseases in the poultry industry and may threaten human health due to its potential zoonosis. In this study, we confirmed that *clbG* was necessary for the APEC XM strain to produce colibactin. The deletion of *clbG* on APEC XM contributed to lowered γH2AX expression, no megalocytosis, and no cell cycle arrest in vitro. None of the 4-week Institute of Cancer Research mice infected with the APEC XM Δ*clbG* contracted meningitis or displayed weakened clinical symptoms. Fewer histopathological lesions were observed in the APEC XM Δ*clbG* group. The bacterial colonization of tissues and the relative expression of cytokines (*IL-1β*, *IL-6*, and *TNF-α*) in the brains decreased significantly in the APEC XM Δ*clbG* group compared to those in the APEC XM group. The tight junction proteins (claudin-5, occludin, and ZO-1) were not significantly destroyed in APEC XM Δ*clbG* group in vivo and in vitro. In conclusion, *clbG* is necessary for the synthesis of the genotoxin colibactin and affects the development of APEC meningitis in mice.

## 1. Introduction

In 2006, colibactin was identified in a neonatal meningitis *E. coli* (NMEC) strain (IHE3034) by Nougayrède and colleagues [1]. Colibactin is a natural and genotoxic chemical compound, which is synthesized by a hybrid non-ribosomal peptide synthetase-polyketide synthase (NRPS-PKS) assembly line encoded by the *pks* genomic island. *E. coli*, *Citrobacter koseri*, *Klebsiella pneumoniae*, and *Klebsiella aerogenes* harbor this *pks* genomic island [2]. The *pks* island contains 19 genes (*clbA* to *clbS*), including phosphopantetheinyl transferase (*clbA*), the nonribosomal peptide synthetases (*clbN* and *clbB*), freestanding acyltransferase (*clbG*), prodrug transporter (*clbM*), and colibactin-maturing peptidase (*clbP*). Colibactin has been proven to be related to mammalian cell DNA double-strand breaks (DSBs), chromosome aberrations, and cell cycle arrest in the G2/M phase [1,3]. Our previous work found expression of many genes in *pks* island changed when avian pathogenic *E. coli* (APEC) XM strain infected the bEnd.3 cells [4]. However, most studies of colibactin just focused on cell senescence [5], intestinal homeostasis regulation [6], tumor microenvironment modification [7], and colon tumor growth [8] and limited studies have been undertaken regarding meningitis. Therefore, this study aimed to achieve a better understanding of the contributions of colibactin to *E. coli* in the process of meningitis.

APEC is an important member of the extraintestinal pathogenic *E. coli* (ExPEC) group. APEC causes severe acute systemic disease and leads to extensive economic losses in poultry industries. NMEC is another member of the ExPEC group, and it is the predominant Gram-negative bacterial pathogen associated with meningitis in newborn infants that accounts for high mortality and morbidity rates [9,10]. Increasing evidence indicates that ExPEC infection is more likely linked to the phylogenetic background of one strain than to its ecological background [11,12]. A majority of NMEC [13,14] and highly virulent strains of APEC [15,16] belong to the phylogenetic group B2. In addition, APEC has a broad range of virulence factors, similar to NMEC strains, including outer membrane protein A (OmpA), type I fimbriae (FimH) [17], and ferric aerobactin receptor (IutA) [18], which give APEC more fitness and pathogenic advantages in mammals. Previous studies demonstrated that APEC can cause bacteremia, sepsis, and meningitis in neonatal rats or mice [19,20]. ExPEC strains isolated from humans and fowl have a high genetic similarity, and some of them can be transmitted between humans and avians [21,22]. Due to the high plasticity of the *E. coli* genome, APEC strains are speculated as an armory of NMEC and have the ability to be potential zoonotic bacteria. 

The colibactin-producing bacteria could profoundly affect their host’s health. The rates of *pks*^+^ *E. coli* isolated from healthy neonates’ stools [23] or grown-up guts [24] are lower than 35%. However, the percentages of *pks*^+^ *E. coli* increased noticeably in extraintestinal infections, such as urinary tract infection [25], sepsis, pneumonia, and neonatal meningitis [26]. *Pks* island is strongly associated with *E. coli* of phylogroup B2 [2,23], which is the most common phylogroup of NMEC (67.92–78.8%) [27,28] and is related to the high pathogenicity of APEC [15,16]. The rate of *E. coli* carrying *pks* islands is also frequently associated with many important virulence factors for meningitis, such as adhesins, hemolysins, toxins, and siderophores [2]. In previous studies, colibactin could induce apoptosis of T lymphocytes, and has been involved in systemic infection and meningitis in mouse models [29]. A *pks^+^ K. pneumoniae* infected BALB/c mouse model showed that colibactin played a key role in pathogenic steps toward the development of meningitis [30]. However, the contribution of colibactin to *E. coli* meningitis is still unknown. As the mature colibactin is still difficult to extract or purify from bacteria to date [31], it is difficult to investigate the role of pure colibactin in meningitis development. *clbG* belongs to the *pks* island and is the only component to recognize the aminomalonyl-acyl carrier protein (AM-ACP) extender unit and transfer it to multiple polyketide synthase (PKS) modules [32]. In this study, we deleted the *clbG* gene to reduce colibactin production in APEC XM, and established a meningitis mouse model to find out the contribution of *clbG* in the synthesis of colibactin and the development of meningitis.

## 2. Results

### 2.1. Genetic Stability and Growth Curves of the Deletion and Complemented Mutants

**Figure 1:**
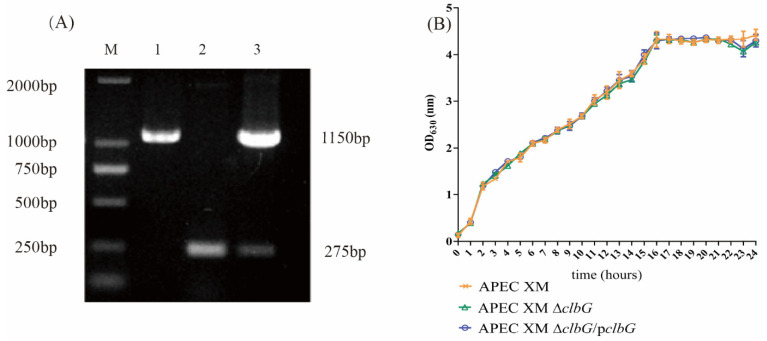
Verification of mutants and the ability of reproduction. (**A**) Identification of mutants by PCR. The avian pathogenic *Escherichia coli* (APEC) XM, APEC XM Δ*clbG*, and APEC XM Δ*clbG*/p*clbG* were amplified with primer P1/P2. (Lane M, DNA molecular size marker; Lane 1, APEC XM; Lane 2, APEC XM Δ*clbG*; and Lane 3, APEC XM Δ*clbG*/p*clbG*) (**B**) Growth curves of bacteria. APEC XM, APEC XM Δ*clbG*, and APEC XM Δ*clbG*/p*clbG* were grown in LB broth at 37 °C under aerobic conditions. The absorbance of culture at OD_630_ was measured per hour. The growth curves were presented as the mean ± standard errors of the mean for three independent experiments. There was no difference in growth between the three strains.

The deletion (APEC XM Δ*clbG*) and complemented (APEC XM Δ*clbG*/p*clbG*) mutants were successfully constructed (Figure 1A). DNA sequencing identified APEC XM Δ*clbG* and APEC XM Δ*clbG*/p*clbG* without any spurious mutation after 30 generations. The growths of the deletion and complemented mutants were similar to that of APEC XM in the LB broth media (Figure 1B). The result supported the fact that the deletion of *clbG* did not affect the growth rate.

### 2.2. Deletion of clbG Affects the Colibactin Production of Avian Pathogenic Escherichia coli (APEC) XM

**Figure 2:**
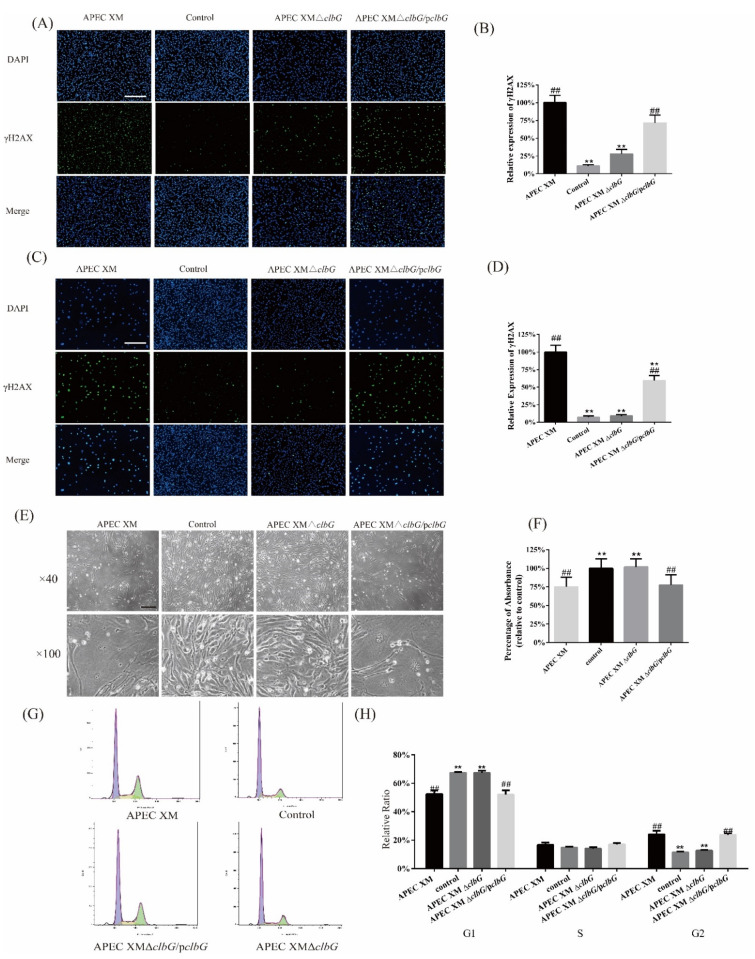
*ClbG* is required for colibactin synthesis and genotoxic effects of APEC XM on bEnd.3 cells. BEnd.3 cells were infected with a multiplicity of infection (MOI) of 100 bacteria/cell for 4 h in the APEC XM*,* APEC XM Δ*clbG* or APEC XM Δ*clbG*/p*clbG* group. For the control group, cells were not infected with any bacteria for 4 h. After 4 h treatment, the bEnd.3 cell DNA double-strand breaks induced by colibactin were assayed by immunofluorescence staining of γH2AX at 0 h post-incubation (hpi) (**A**). The nuclear DNA and γH2AX are colored in blue and green, respectively (bar = 200 μm). The percentage of γH2AX positive cells within the total population of cells at 0 hpi (**B**) are shown in bar graphs. The ratio of the APEC XM group was set as 100%. After the same treatment, the bEnd.3 cell DSBs induced by colibactin were assayed by immunofluorescence staining γH2AX (**C**) and quantified (**D**) of γH2AX positive cells at 72 hpi. (**E**) Megalocytosis induced by colibactin was observed at 72 hpi by an inverted microscope (bar = 100 μm). APEC XM and APEC XM Δ*clbG*/p*clbG* induced a progressive enlargement of the bEnd.3 cell body and the nucleus. (**F**) The megalocytosis of methylene blue stained cells was quantified by the absorbance values at 630 nm by a microplate reader. The data of the control group was set as 100%. (**G**) The cell cycles of bEnd.3 cells were assayed by flow cytometry (purple, G0/G1 phase; yellow, S phase; green, G2/M phase). APEC XM and APEC XM Δ*clbG*/p*clbG* resulted in G2/M phase accumulation in infected cells (**H**) Quantification analyses of cell-cycle distribution (G1 phase, S phase, and G2 phase) of bEnd.3 cells. The data were analyzed with one-way analysis of variance (ANOVA) and presented as the mean ± standard errors of the mean for three independent experiments. (**, *p* < 0.01, versus APEC XM group; ##, *p* < 0.01, versus control group).

To confirm the role of *clbG* during the colibactin biosynthesis process, we detected the γH2AX expression, megalocytosis level, and cell cycle phases in infected bEnd.3 cells to quantify the colibactin production of each strain. Colibactin induces an increase in γH2AX expression of infected cells due to DSBs. The immunofluorescence staining assay of γH2AX was used to measure the level of DSBs in bEnd.3 cells. After 4 h of infection, the percentages of γH2AX positive cells in the APEC XM group and the APEC XM Δ*clbG*/p*clbG* group increased significantly at 0 h post-incubation (hpi, Figure 2A,B) and 72 hpi (Figure 2C,D), compared with those in the control group. There was no difference between the APEC XM Δ*clbG* group and the control group at 0 hpi and 72 hpi. 

Meanwhile, colibactin led to a progressive enlargement of bEnd.3 cell body and nucleus due to blocking of the cell cycle. The absorbance values of methylene blue staining cells at 630 nm were used to quantify the cytotoxicity. As expected, the cells infected by AEPC XM and APEC XM Δ*clbG*/p*clbG* showed similar enhanced megalocytosis (Figure 2E) and significantly lower absorbance (630 nm) (Figure 2F), compared with the control group. The cells infected with APEC XM Δ*clbG* displayed a normal cell morphology and a similar absorbance as the control group. When the DNA damage was beyond repair, cells underwent cycle arrest at G2/M. The cell cycle analysis was tested by flow cytometry. As shown in Figure 2G,H, the APEC XM and APEC XM Δ*clbG*/p*clbG* resulted in G2/M phase accumulation in bEnd.3 cells. APEC XM Δ*clbG* did not induce cell cycle arrest (Figure 2G). The cell-cycle distribution (G1 phase, S phase, and G2 phase) of bEnd.3 cells in the APEC XM Δ*clbG* group were similar to the control group (Figure 2H).

The results for the γH2AX expression, megalocytosis level, and cell cycle phases in bEnd.3 cells confirmed that the deletion of *clbG* reduced the colibactin production of APEC XM. It also supported the fact that APEC XM Δ*clbG*/p*clbG* restored the genotoxicity of colibactin.

### 2.3. ClbG Is Required for the Pathogenicity of APEC XM In Vivo

**Figure 3:**
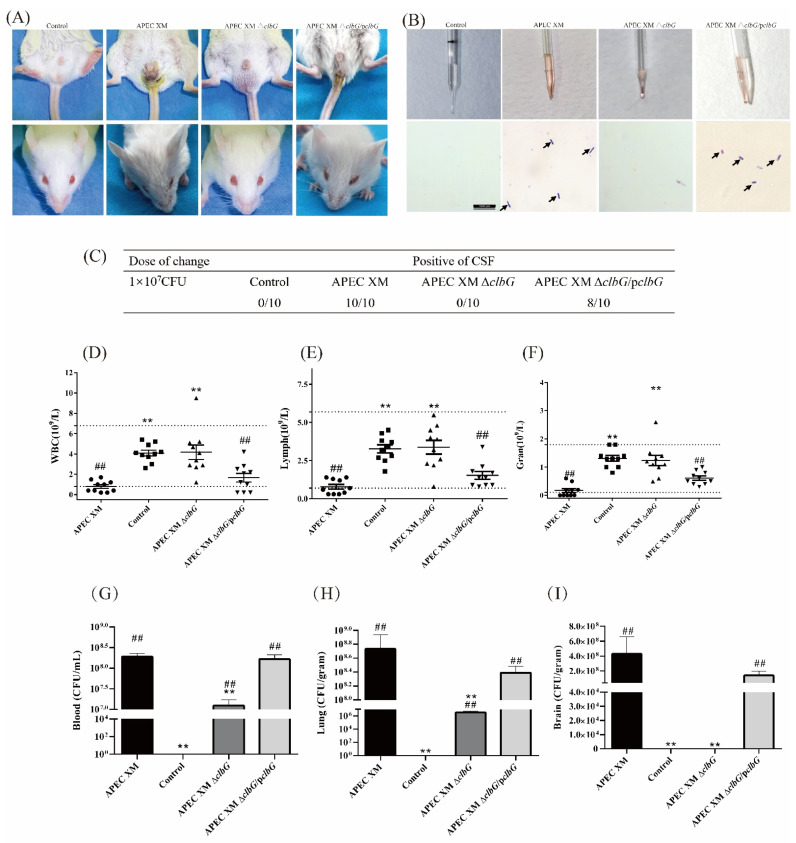
*ClbG* affects pathogenicity of APEC XM on 4-week Institute of Cancer Research (ICR) mouse. Four-week-old ICR mice were inoculated intraperitoneally with a dose of 10^7^ CFU. *E. coli* in 100 μL saline or an equal volume of sterile saline. (**A**) The representative images of mice in each group. The mice in APEC XM group and the APEC XM Δ*clbG*/p*clbG* group displayed lackluster coat, eyelid closure with thick red ocular discharge (lower lane), and diarrhea (upper lane). (**B**) The physical examination (upper lane) and Diff-Quik staining (lower lane) of cerebrospinal fluid (CSF) samples. *E. coli* (Black arrow) were found in the CSF samples of the APEC XM group and the APEC XM Δ*clbG*/p*clbG* group (bar = 10 μm). (**C**) The presence of *E. coli* in CSF staining is the positive standard of meningitis. All mice in the APEC XM group and 80% mice in the APEC XM Δ*clbG*/p*clbG* group suffered meningitis, whereas no mice suffered meningitis in the APEC XM Δ*clbG* or the control group. (**D**–**F**) Complete blood counts (CBC) test. The CBC data for white blood cells (**D**), lymphocytes (**E**), and neutrophils (**F**) are shown in the scatter plot. The dotted lines of the scatter plot charts represent the normal ranges of the cell population. The absolute counts of white blood cells, lymphocytes, and neutrophils declined in the APEC XM group and the APEC XM Δ*clbG*/p*clbG* group. (**G**–**I**) Bacterial load assay for lung, blood, and brain. Calculation of bacteria from blood (**G**), lung (**H**), and brain (**I**) in each group by plate counting. No bacterium was isolated from brain tissue samples in the control group nor the APEC XM Δ*clbG* group. The results were analyzed with one-way ANOVA and presented as the mean ± standard errors of the mean for three independent experiments. (**, *p* < 0.01, versus APEC XM group; ##, *p* < 0.01, versus control group).

The effect of the *clbG* gene on pathogenicity was evaluated using Institute of Cancer Research (ICR) mice by intraperitoneal injection of APEC XM, APEC XM Δ*clbG*, and APEC XM Δ*clbG*/p*clbG*, respectively. The health status of the mice was assessed by a clinical score as described previously [33,34]. When mice of the APEC XM group were no longer able to walk (clinical score 3), mice of four groups were sacrificed and none of the mice died spontaneously. The mice showed depression, rough fur, eyelid closure with thick red-eye discharge, diarrhea, and neurological symptoms after the challenge with APEC XM (Figure 3A, clinical score 3). The mice in APEC XM Δ*clbG*/p*clbG* group presented a sign of eyelid closure with thick eye discharge and diarrhea (Figure 3A, clinical score 2–3). In the APEC XM Δ*clbG* group or the control group, there were very mild or no symptoms (Figure 3A, clinical score 0). The diff-quik dye of the cerebrospinal fluid (CSF, Figure 3B) demonstrated all mice in the APEC XM group and most mice (8/10) in APEC XM Δ*clbG/*p*clbG* group suffered meningitis (Figure 3C). None of the mice suffered meningitis in the APEC XM Δ*clbG* or the control group. The absolute counts of white blood cells (Figure 3D), lymphocytes (Figure 3E), and neutrophils (Figure 3F) in the APEC XM group and APEC XM Δ*clbG/*p*clbG* group were lower than those in the control group. There were no significant differences between the APEC XM Δ*clbG* and control group.

The bacterial loads in the blood (Figure 3G) and lung (Figure 3H) decreased significantly in the APEC XM Δ*clbG*, compared with the APEC XM group. The bacterial loads of the lung and blood in the APEC XM Δ*clbG*/p*clbG* group were similar to those in the APEC XM group. Importantly, no bacterium was isolated from brain tissue samples in the APEC XM Δ*clbG* group or the control group (Figure 3I). 

The results of ICR mouse infection indicated that *clbG* is necessary for pathogenicity of APEC XM in vivo.

### 2.4. Relative Cytokines Profiles and Histopathological Findings in Mouse Brains

**Figure 4:**
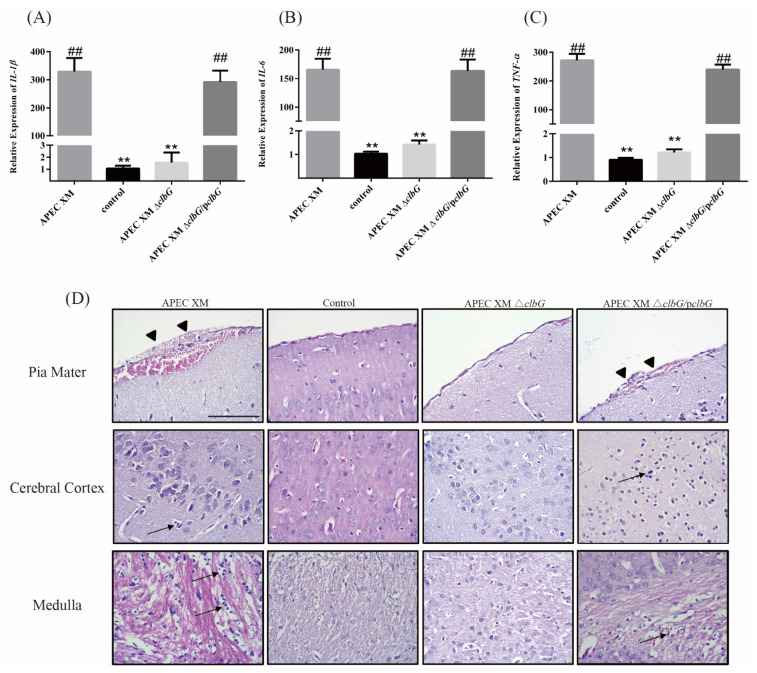
*ClbG* contributes to inflammation responses and brain damage in mice meningitis. Four-week-old ICR mice were inoculated intraperitoneally with a dose of 10^7^ CFU. *E. coli* in 100 μL of saline or an equal volume of sterile saline for 12 h. (**A–C**) The mRNA transcripts levels of *IL-1β* (**A**), *IL-6* (**B**), and *TNF-α* (**C**) were determined by qRT-PCR from mouse brains of each group (normalized to *GAPDH*). The results were analyzed with one-way ANOVA and presented as the mean ± standard errors of the mean for three independent experiments. (**, *p* < 0.01, versus APEC XM group; ##, *p* < 0.01, versus control group) (**D**) Histopathological analysis of brain tissue. Representative hematoxylin-eosin staining images of pia mater (upper lane), cerebral cortex (middle lane), and medulla (lower lane) of brain sections (bar = 100 μm). Leukocyte infiltration (Black Arrow) was observed in the pia mater, cerebral cortex, and medulla. The thickened pia mater with hemorrhage (Black Arrowhead) was observed in the brain section of the APEC XM group and the APEC XM Δ*clbG*/p*clbG*.

The relative expression levels of *IL-1β*, *IL-6*, and *TNF-α* mRNA in brain tissues were measured by qRT-PCR. The relative expression of *IL-1β* (Figure 4A), *IL-6* (Figure 4B), and *TNF-α* (Figure 4C) mRNA were similar between the APEC XM group and the APEC XM Δ*clbG*/p*clbG* group, and both of them were significantly increased compared with the control group. No significant differences were found between APEC XM Δ*clbG* and the control group in the relative expression of these cytokines. 

The histopathological analysis showed a marked thickening and hemorrhage of the pia mater (Figure 4D, black arrowhead), and moderate leukocyte infiltration in the pia mater, cerebral cortex, and medulla in the APEC XM group (Figure 4D, black arrow). Also, a slight hemorrhage in the medulla and a marked thickening and hemorrhage of the pia mater (Figure 4D, black arrowhead) were found in the APEC XM Δ*clbG*/p*clbG* group. The histological changes mentioned above were not found in the APEC XM Δ*clbG* or the control group.

The results of relative cytokines profiles and histopathological findings indicated that *clbG* is necessary for the pathogenicity of APEC XM in vivo.

### 2.5. ClbG Contributes to the Disruption of the Blood–Brain Barrier In Vitro and In Vivo 

**Figure 5:**
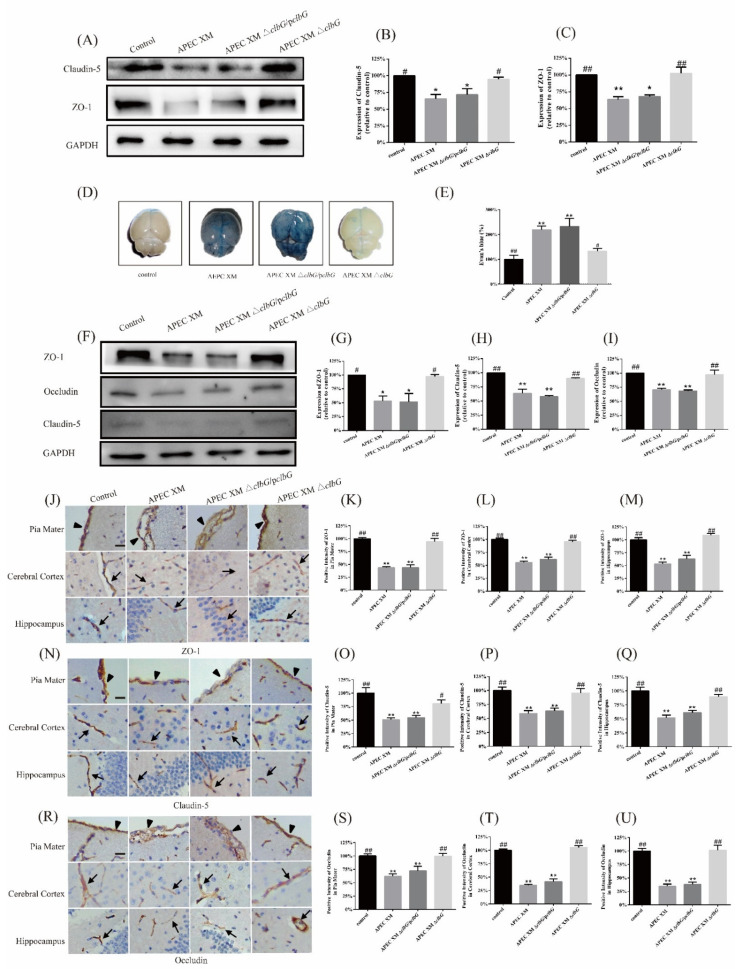
*ClbG* contributes to the disruption of the blood–brain barrier. (**A**–**C**) The expression of claudin-5 and ZO-1 in vitro. BEnd.3 cells were infected with a multiplicity of infection (MOI) of 100 bacteria/cell for 4 h in the APEC XM*,* APEC XM Δ*clbG* and APEC XM Δ*clbG*/p*clbG* group in the following experiments. Cells without infection of bacteria were set as the control group. Blots (**A**) and relative protein levels of claudin-5 (**B**) of and ZO-1 (**C**), normalized to GAPDH. The data of the control group was set as 100%. ZO-1 and claudin-5 proteins decreased significantly in the APEC XM group and APEC XM Δ*clbG*/p*clbG* group, compared to the control group. (**D**–**U**) Four-week-old ICR mice were inoculated intraperitoneally with a dose of 10^7^ CFU *E. coli* in 100 μL saline or an equal volume of sterile saline for 12 h. (**D**) The representative bright-field photographs of Evans blue stained mouse brains were taken in dorsal view. (**E**) The amount of Evans blue dye was quantified by measuring the absorbance (OD_630_) after brain homogenization and precipitation. The data of the control group were set as 100%. Increase of blood–brain barrier permeability was found in the APEC XM group and APEC XM Δ*clbG*/p*clbG* group, but not in APEC XM Δ*clbG* group. (**F**–**I**) The expression of claudin-5, occludin, and ZO-1 in vivo was measured by Western blotting. Representative Western blots (**F**) and the relative protein levels of and ZO-1 (**G**), claudin-5 (**H**), and occludin (**I**), normalized to GAPDH. The data of the control group was set as 100%. ZO-1, occludin, and claudin-5 proteins decreased significantly in the APEC XM group and the APEC XM Δ*clbG*/p*clbG* group, compared to the control group. (**J**–**U**) The expression of tight junction proteins in vivo was also measured by immunohistochemistry (IHC). The representative IHC pictures of ZO-1 (**J**), claudin-5 (**N**), and occludin (**R**) protein levels in the pia mater, cerebral cortex, and hippocampus of brain sections. The black arrowheads pointed to the pia maters and the black arrows pointed to the capillaries (bar = 20 μm). Graphical representations of the relative optical densities of ZO-1 (**K**–**M**), claudin-5 (**O**–**Q**), and occludin (**S**–**U**) in pia mater, cerebral, and hippocampus, respectively. The data of the control group was set as 100%. The results were analyzed with one-way ANOVA and presented as the means ± standard errors of the mean for three independent experiments. (**, *p* < 0.01, *, 0.01 < *p* < 0.05 versus APEC XM group; ##, *p* < 0.01, #, 0.01 < *p* < 0.05 versus control group).

Western blotting was used to measure the expression of ZO-1 and claudin-5 protein in vitro. The levels of claudin-5 (Figure 5A,B) and ZO-1(Figure 5A,C) decreased significantly in the APEC XM group and the APEC XM Δ*clbG*/p*clbG* group, compared to the control group. The expression of claudin-5 and ZO-1 in the APEC XM Δ*clbG* group was higher than those in the APEC XM group.

Evans blue dye analysis was performed to measure the integrity and permeability of the blood–brain barrier (BBB) in vivo. The brain from APEC XM infected mouse was stained blue by Evans blue dye (Figure 5D). The extravasation of Evans blue dye from the brain in the APEC XM Δ*clbG* and the control group were significantly lower than those in the APEC XM group and the APEC XM Δ*clbG*/p*clbG* group (Figure 5E). 

Then, Western blotting and immunohistochemical staining were used to measure the tight junctional protein expression in vivo. The expression of ZO-1 (Figure 5F,G), claudin-5 (Figure 5F,H), and occludin (Figure 5F,I) decreased significantly in the APEC XM group and the APEC XM Δ*clbG*/p*clbG* group, compared with the control group. There were no significant differences in the expression of ZO-1, claudin-5, and occludin between the APEC XM Δ*clbG* group and the control group. 

The immunohistochemical staining showed the expression of ZO-1 (Figure 5J), claudin-5 (Figure 5N), and occludin (Figure 5R) in brain sections per group. The relative optical density of ZO-1 in pia mater (Figure 5K), cerebral (Figure 5L), and hippocampus (Figure 5M) from the APEC XM group and the APEC XM Δ*clbG*/p*clbG* group decreased significantly, compared to the control group. The relative optical densities of claudin-5 and occludin in the APEC XM group and the APEC XM Δ*clbG*/p*clbG* group showed similar expression patterns. All the detected tight junctional proteins in pia mater, cerebral, and hippocampus of brain sections from the APEC XM Δ*clbG* group were similar to those from the control group.

Based on the results above, the deletion of *clbG* contributes to the disruption of the BBB by APEC XM in vitro and in vivo.

## 3. Discussion

*E. coli* is the most common Gram-negative bacteria that causes newborn meningitis and leads to over 10% of mortality [35,36] and approximately 50% of neurological disabilities [37] in newborn infections. *E. coli* has many important virulence factors [38] and bacterial genotoxins [39] to survive in the bloodstream and invade the meninges of newborns. Colibactin was first found in an NMEC strain (IHE3034) [1], and the mRNA levels of *pks* island genes changed in in vitro infection of APEC XM in our previous work [4]. Most studies of colibactin have focused on gut homeostasis or colorectal cancer, but limited studies have been undertaken regarding meningitis. In this study, we established a 4-week ICR mouse meningitis model to find out the contribution of *clbG* in the development of meningitis. The results indicated that *clbG* was a necessary component for the synthesis of genotoxic colibactin and impact the development of meningitis in mouse.

Freestanding acyltransferase (AT, ClbG) is an important member of the PKS unit in *pks* island [1]. AM-ACP incorporation is critical for constructing genotoxic metabolites of colibactin [1]. The ^14^C gel autoradiography assay confirmed that ClbG was the only component in the *pks* assembly line to recognize the AM-ACP extender unit and transfer AM-ClbE to multiple PKS modules, including PKSs that lack functional AT domains (ClbC, ClbK, and ClbO) and a cis-AT PKS (ClbI) [32]. Therefore, ClbG is necessary for genotoxin production [40]. In line with previous studies of genes in the *pks* island (such as *clbA* [30] or *clbP* [26]), deletion of *clbG* repressed colibactin production and reduced genotoxicity to the bEnd.3 cells in vivo, which indicated that *clbG* was a key gene for producing colibactin in APEC XM strain. The results of colibactin cytotoxicity assays also showed that APEC XM Δ*clbG*/p*clbG* restored the genotoxicity. In addition, the deletion of *clbG* affected the pathogenicity of newborn ICR mice in vivo as observed in this study. After the invasion of the host, pathogens need to rapidly adjust to the microenvironments of different tissues and colonize in organs to cause bacteremia and systemic infections [30]. A high degree of bacteremia is a prerequisite for *E. coli* to result in meningitis [41,42]. Unlike the high degree of organ colonization in the APEC XM group, the bacterial loads in the APEC XM Δ*clbG* group decreased in the lungs and blood. Colibactin may not only act as a classical virulence factor facilitating bacterial translocation and survival [43], but also as a fitness factor, improving the colonization of the gastrointestinal tract by slowing the renewal of enterocytes [23]. Colibactin induced a decrease of regulatory T cell (CD4^+^CD25^+^ FoxP3^+^) populations [44] in the newborn rat early colonized by *pks*^+^ *E. coli*. Meanwhile, APEC XM induced a decrease of lymphocytes in meningitic mice, which was alleviated in APEC XM Δ*clbG* infected mice. The decrease in lymphocytes was similar to previous experimental *pks*^+^ septicemic mice, and the survival rate of mice or rats in sepsis and meningitis induced by *pks*^+^ *E. coli* decreased [26,29]. After the invasion of the blood compartment, the bacteria entered the central nervous system, and colonization of the brain is another key step to meningitis development [37]. In histopathological analysis, brain injuries including necrotic cortical injury and apoptotic hippocampal injury [45,46] were found in the *E. coli* meningitis mice. All these histopathological signs, such as the severe thickening of the pia mater, leukocyte infiltration into brain tissue and hemorrhage shown in the APEC XM group, were not found in the APEC XM Δ*clbG* group. The results mentioned above indicate that *clbG* is a necessary part of the synthesis of genotoxic colibactin, which is also strongly related to meningitis induced by APEC XM. To date, it is the first study to demonstrate the direct pathogenicity of *clbG* in vivo.

In the pathogenesis of cerebral injury in bacterial meningitis, TNF-α, IL-6, and IL-1β are major early response cytokines that trigger, often in synergy, a cascade of inflammatory mediators, including other cytokines, oxygen intermediates, and chemokines [47]. These cytokines are produced by multiple brain cell types, such as cerebromicrovascular endothelial cells [48], astrocytes [49], and microglia [50]. The mRNA expression of *TNF-α*, *IL-6*, and *IL-1β* was markedly up-regulated in the pneumococcal [51,52] or *E. coli* meningitis [53,54,55] rodent model, similar to the relative expression of detected cytokines in the APEC XM group. The overproduction of cytokines in the brain could trigger immune system hyperactivity and induce a “cytokine storm” damaging the central nervous system. IL-1β contributes to macrophage recruitment, *Streptococcus pneumoniae* clearance [56], and the protection of mice from lethal Gram-negative bacterial infection [57]. IL-1β also induces the expression of many other cytokines [58] and growth factors [59], such as TNF-α and IL-6. TNF-α is another important early-response cytokine and is related to a fatal outcome in meningitis [60]. However, TNF-α deficiency did not affect the brain bacterial titers and leukocyte recruitment into the subarachnoid space in the mouse model of central nervous system infection in one study [61]. Both IL-1β and TNF-α are bone marrow stimulants that grow in a number of myeloid progenitors and promote the recruiting of neutrophils in the inflammation site [60]. IL-6 is a pleiotropic cytokine with both pro-inflammatory and anti-inflammatory effects. It appears to be a good marker of severity during bacterial infection [62]. IL-6 deficiency caused an increased leukocyte concentration in CSF of pneumococcus-infected mice. IL-6 appears to control the inflammatory response by down-regulating the response of chemokines and/or proinflammatory cytokines [47]. In addition, these inflammatory cytokines play a role in BBB integrity. IL-17, IL-6, and/or TNF-α, alone or in combination, trigger an inflammatory reaction, which leads to a significant increase of permeability and a decrease of tight junction proteins (ZO-1 and claudin-5) in bEnd.3 BBB model [63]. Interestingly, TNF-α was shown to exert different effects on tight junction proteins disruption in different organs. For example, TNF-α reduced occludin and ZO-1 expressions in pulmonary microvascular endothelial cells [64] and hCMEC/D3 cells [65]. One study reported that the response of TNF-α and IL-β induced leak in human dermal microvascular cells in two distinct NF-κB-dependent steps, the first involving Rho-associated kinase and the second likely to involve an as yet unidentified but structurally related protein kinase(s) [66]. In a co-culture model, consisting of human brain microvascular endothelial cells and pericytes, changes in claudin-5 localization and expression after TNF-α treatment and recovery time were dependent on TNF-α concentration [67]. In the meantime, IL-6 increased endothelial permeability and produced ZO-1 mislocalization, actin structure remodeling, and increased actin contractility [68]. APEC XM Δ*clbG* did not increase the relative mRNA expressions of *TNF-α*, *IL-6*, and *IL-1β* in diseased mice, which might be a reason for the relative integrity of the BBB in this group. However, more studies are needed to elucidate the mechanism of tight junction proteins degradation by *clbG*.

BBB regulates the transport of various molecules and maintains brain homeostasis. The disruption of BBB is a hallmark event in the pathophysiology of bacterial meningitis [69]. Structurally, the inter-endothelial tight junction complexes comprising occludin, claudins, and membrane-directed scaffolding proteins (such as zonula occludentes-1, ZO-1) contribute to the physical barrier nature of BBB and strictly limit the molecular/cellular influx from circulation [70,71]. ZO-1, claudin-5, and occludin decreased at the transcript and/ or protein level of brain endothelial cells after *Group B Streptococcus* [72,73,74], *Neisseria meningitidis* [75], *Streptococcus suis* [76], or *E. coli* [77] infection. In vitro, the disruption of tight junction proteins in bEnd.3 cells were alleviated in the APEC XM Δ*clbG* group, as well as an in vivo mouse model by immunohistochemistry and Western blotting assays. The vascular leakage was also reduced in the APEC XM Δ*clbG* group. Therefore, *clbG* might play an important role in disordering the tight junction proteins of BBB. ZO-1 was first identified as a tight junction-associated protein and expressed on both EC and epithelial cell surfaces [78]. It serves as a scaffold for tight junction formation by binding to the C-terminal of the cytoplasmic tail of occludin and the cytoskeletal protein spectrin. When ZO-1 expression is reduced or dissociated from membrane proteins, the BBB stringency is compromised. Occludin, a 65-kD protein, was the first tight junction-associated transmembrane protein identified and was related to cytoskeletal signaling proteins (ZO-1 and ZO-2) [79]. The barrier function of occludin depends on its special structure domains. To date, multiple factors, such as matrix metalloproteinases (MMPs)-dependent degradation [80], phosphorylation [81], ubiquitination [82], and other cytokines [83] (IL-1β, TNF-α, and IFN-γ), have been proven to regulate occludin functions on BBB permeability. Claudin-5 is present in both human and mouse early fetus brain vessels and continues to increase during postnatal development for BBB maturation [84,85]. Claudin-5 is observed as a key component of the tight junction strand for selectively decreasing the permeability to ions, particularly in brain endothelial cells. Significantly, the conserved cysteines of claudin-5 were crucial for maintaining high transepithelial electrical resistance [86]. The dysfunction of claudin-5 protein was related to endothelial permeability in a series of pathological processes, including inflammation, tumor edema [87], toxic damage [88], and high glucose damage [89].

## 4. Conclusions

In summary, our results showed that *clbG* is a key virulence factor for APEC XM to induce meningitis. *clbG* contributed to the disruption of tight junction proteins expression, and the increase in the inflammatory response in vivo and in vitro infection. The experimental result indicated that the blockage of the colibactin production, through the deletion of *clbG*, substantially hindered APEC XM survival in the bloodstream. The results of the mouse pathogenicity test suggested that APEC XM may take advantage of blood to gain access to the central nervous system, which might require the involvement of colibactin.

## 5. Materials and Methods

### 5.1. Ethics Statement

The animal experiments followed the National Institute of Health guidelines for the ethical use of animals in China. All procedures were approved by the Animal Care and Ethics Committee of Yangzhou University. Four-week-old ICR mice were provided by the Comparative Medicine Center of Yangzhou University (License number: SCXK (Su) 2017-0007). All mice had free access to food and water under a 12 h light/dark cycle and were monitored twice a day. The tissue samples were collected in mice under isoflurane anesthesia. The mice were finally euthanized via isoflurane anesthesia to minimize the suffering.

### 5.2. Bacterial Strains, Growth Conditions, and Plasmids

The APEC XM strain (O2: K1, donated by Dr. Guoqiang Zhu, Yangzhou University) was isolated from the brain of a duck with meningitis and septicemia. APEC XM strain grew on Luria–Bertani (LB) plates or in LB broth (180 rpm/min) at 37 °C aerobically. The culture medium and agar plates, with the addition of ampicillin (100 μg/mL) or chloramphenicol (34 μg/mL), as appropriate. The strains and plasmids used in this study were listed in Table 1.

### 5.3. Construction of clbG Deletion and Complemented Mutants

Bacteriophage λ Red recombinase system was used to inactivate the *clbG* of APEC XM strain with the primers P3/P4 and plasmid pKD3, pKD46, and pCP20 as described previously [90] (Table 1). The full length of the *clbG* gene was cloned into plasmid pACYC184 and the recombinant plasmid pACYC184-*clbG* was transformed into *clbG*-deletion mutant for constructing the complemented mutant. The deletion and complemented mutants were confirmed by the combination of PCR and DNA sequencing. All primers and plasmids used in this study are listed in Table 1.

### 5.4. Growth Curves

Generally, 200 μL (1 × 10^8^ CFU) bacteria in the mid-log phase were inoculated in LB medium with or without ampicillin at 37 °C with 180 rpm/min shaking. Then, 200 μL samples were taken every 1h and detected at 630 nm using a spectrophotometer (BioTek ELx800, Winooski, VT, USA) for the optical density, until the bacteria grew at a static phase. The above experiments were repeated three times independently. The growth curves were drawn by GraphPad Prism 5.0 software (GraphPad Software, San Diego, CA, USA).

### 5.5. Cell Culture

The bEnd.3 cells (ATCC CRL-2299, American Type Culture Collection, Manassas, VA, USA) were cultured in the DMEM (Invitrogen, Carlsbad, CA, USA), supplemented with 10% heat-inactivated fetal bovine serum (FBS; Gibco, Carlsbad, CA, USA) in a humidified incubator at 37 °C with 5% CO_2_.

### 5.6. Colibactin Cytotoxicity Assays

The bEnd.3 cells (about 75% confluence) were infected with APEC XM, APEC XM Δ*clbG*, and APEC XM Δ*clbG*/p*clbG* with a MOI of 100, respectively. After 4 h infection, bEnd.3 cells were washed three times with PBS, and further incubated in DMEM with 10% FBS containing gentamicin (100 μg/mL) until the next treatment.

Colibactin induces an increase in the γH2AX protein of infected cells due to DSBs. The immunofluorescence was used to find the γH2AX expression of infected cells at 0 hpi and 72 hpi [1,91]. The cells were infected and fixed as described above. After that, the cells were permeabilized with 0.1% Triton X-100 for 20 min and processed for immunofluorescence following a standard protocol [92]. The primary antibody was a monoclonal rabbit anti phosphorylated H2AX (#9718, Cell Signaling Technology). The secondary antibody was a goat-anti-rabbit IgG (H + L) Alexa Fluor Plus 488 (A-21070, ThermoFisher Scientific). The cells were stained with 4′,6-diamidino-2-phenylindole (DAPI; Beyotime, Shanghai, China). Finally, the GFP fluorescence was detected and photographed by fluorescence microscope (Leica TCS SP8; Leica Corp., Wetzlar, Germany).

To visualize the colibactin-inducing megalocytosis, the cells were incubated for 72 hpi containing gentamicin. Then, the cells were fixed with 4% paraformaldehyde and stained with 0.1% methylene blue for 20 min. The cells were observed by an inverted microscope. The cytotoxic effects of each strain were quantified by the absorbance values at 630 nm by a microplate reader (BioTek ELx800, Winooski, VT, USA).

Under the same infection condition, the cell cycle of bEnd.3 cells was detected at 48 hpi by the flow cytometer [1,91]. The cells were collected and washed with PBS three times. Then, cells were resuspended in 70% ice-cold ethanol at 4 °C overnight for fixation. The cells were centrifuged at 800 g for 10 min at 4 °C and washed with PBS. FxCycle™ PI/RNase staining solution (F10797, Thermo Fisher Scientific, Waltham, MA, USA) was used to stain cells at room temperature for 15 min and analyzed for DNA content. Data were acquired on a BD LSRFortessa™ flow cytometer (BD Biosciences, Franklin Lakes, NJ, USA) with 10,000 events/determination and analyzed with Flowjo software (Tree Star Inc., Ashland, OR). The experiments were repeated three times independently.

### 5.7. E. coli Meningitis Mouse Model

**Figure 6:**
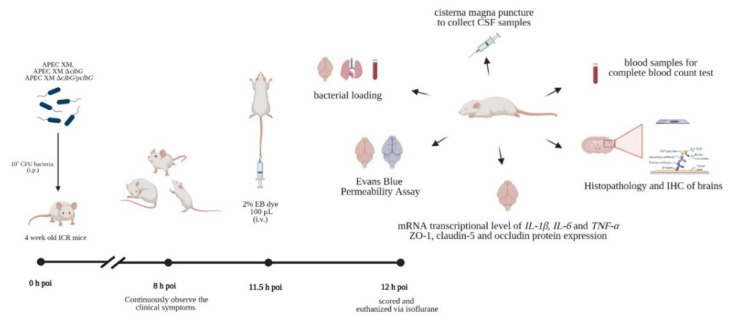
Schematic of mice infection experiments workflow.

The mouse *E. coli* meningitis model was established as previously described [93] (Figure 6). Forty 4-week-old ICR mice were randomly divided into four groups, and each group included 10 mice (five males and five females). The mice were inoculated intraperitoneally with a dose of 10^7^ CFU *E. coli* in 100 μL saline or an equal volume of sterile saline. The clinical symptoms of the mice were observed after 8h poi. The health status of the mice was assessed by a clinical score (0, no apparent behavioral abnormality; 1, moderate lethargy (apparent decrease of spontaneous activity); 2, severe lethargy (rare spontaneous movements, but walking after stimulation by the investigator); 3, unable to walk; 4, dead) [33,34]. When a mouse was no longer able to walk (clinical score 3), it was sacrificed for ethical reasons. None of the animals died spontaneously. CSF samples were collected by cisterna magna puncture under isoflurane anesthesia at 12 h poi. The blood samples were obtained and treated with dipotassium ethylenediaminetetraacetic acid (EDTA K_2_) for the next complete blood count test on an automatic blood cell analyzer (Mindray, BC-1900, Shenzhen, China). The brain samples were collected, frozen in liquid nitrogen instantly, and then stored at −80 °C for subsequent experiments.

### 5.8. Evans Blue (EB) Permeability Assay

For the analysis of changes in BBB permeability in vivo, the mice were injected with 2% EB (100 µL per mouse) via the tail vein. After 30 min, the mice were anesthetized with isoflurane and then perfused with pro-cool PBS to purge the intravascular EB dye. The brains were frozen in the liquid nitrogen, homogenized in 50% trichloroacetic, and incubated at 4 °C for 12 h [94]. The mixture was centrifuged at 15,000× *g* for 30 min at 4 °C to collect the supernatants. The absorbance was measured using a spectrophotometer.

### 5.9. Determination of Bacteria Loadings in the Blood, Lung, and Brain

At 12 h poi, the tissue samples, including lung, blood, and the right hemisphere of the brain, were collected aseptically and homogenized in 1 mL sterile pre-cool PBS. Serial dilutions of each sample were plated on MacConkey agar and cultured at 37 °C for determining CFUs. The bacterial loading was calculated in CFU per gram of brain and lung or per microliter of blood. The PCR test and the agglutination test (K1 and O2) were used to confirm that the colonies were indeed the strain used for infection.

### 5.10. Histopathology of the Brain

At 12 h poi, the mouse brain was fixed immediately in 4% paraformaldehyde for routine processing and paraffin embedding. The embedded samples were cut into 3–4 µm sections by an automated microtome (Leica RM2255, Wetzlar, Germany). Prior to hematoxylin and eosin (H&E) staining, the sections were deparaffinized to remove the embedding medium and rehydrated at room temperature. The stained slices were observed and photographed by a microscope (Nikon, Eclipse 80i, Tokyo, Japan).

### 5.11. Immunohistochemistry of Tight Junction Proteins

The brain sections were prepared as mentioned above. The sections were treated with 3% H_2_O_2_ for 10 min to block endogenous peroxidase activity. Then, they were incubated with citrate buffer for 15 min at 100 °C for antigen retrieval. After blocking with bovine serum albumin (BSA) (Cat#SA1020, Boster Biological Technology, Shanghai, China) at 37 °C for 1h, the sections were incubated with primary antibodies at 4 °C overnight. The primary antibodies used in this study were ZO-1 (1:100; Cat#61-7300, Invitrogen, Co., Ltd., San Diego, CA, USA), occludin (1:100; Cat#71-1500, Invitrogen, Co., Ltd., San Diego, CA, USA) and claudin-5 (1:200; Cat#35-2500, Invitrogen, Co., Ltd., San Diego, CA, USA). Subsequently, the sections were incubated with horseradish peroxidase (HRP) labeling secondary antibody (Cat#SA1020, Boster Biological Technology, Shanghai, China) for 1h and visualized by 3,30-diaminobenzidine tetrahydrochloride (DAB; AR1000, Boster Biological Technology, Shanghai, China). The sections were counterstained with hematoxylin, dehydrated, and viewed under a microscope (Leica TCS SP8; Leica Corp., Wetzlar, Germany). Images were analyzed by the soft Image J.

### 5.12. IL-1β, IL-6, and TNF-α mRNA Expression in Brains

The total RNAs were extracted from homogenized brains using TRIzol solution (Invitrogen Co., Ltd., San Diego, CA, USA) as the manufacturer’s recommendation. RNA purity and quality were measured by nano-drop spectrophotometry. The 900 ng of high-quality RNA was converted into cDNA using the PrimeScript RT reagent Kit with gDNA Eraser (Takara, RR047A, Tokyo, Japan). The qRT-PCR reaction was performed on the CFX CONNECT Real-time PCR machine (Bio-Rad, Louisville, KY, USA) using ChamQ SYBR qRT-PCR Master Mix (2×) (Vazyme, Nanjing, China). The primer sequences of *IL-1β*, *IL-6*, and *TNF-α* are shown in Table 1. The amplification program included an initial denaturation at 95 °C for 10 min, followed by 40 cycles of 95 °C for 30 s, 60 °C for 30 s, and 72 °C for 30 s, and a final extension at 72 °C for 10 min. The relative mRNA expression of each gene was calculated using 2^−ΔΔCt^ with *GAPDH* as the internal reference.

### 5.13. Western Blotting

The RIPA lysate buffer (Beyotime Biotech, Nantong, China) was used to extract total proteins from the brain and the bicinchoninic acid protein assay kit (Beyotime, Shanghai, China) was used to determine the concentrations of proteins. The total proteins were run on SDS-PAGE gels and then blotted onto nitrocellulose membranes (Millipore, Billerica, MA, USA) using the BIO-RAD Mini Trans-Blot^®^ System (Bio-Rad, Louisville, KY, USA). The membranes were incubated with 5% skim milk to reduce nonspecific binding for 1h at room temperature. Then, the membranes were incubated overnight at 4 °C in primary antibodies. The primary antibodies used in this study were ZO-1 (1:1000; Cat#61-7300, Invitrogen), Occludin (1:500; Cat#71-1500, Invitrogen), and Claudin-5 (1:50; Cat#35-2500, Invitrogen) and GAPDH (1:1000; Cat#2118, Cell Signaling Technology). After the membranes were washed four times with TBST, they were incubated with HRP-conjugated secondary antibodies (all at 1:1000 dilution in 5% nonfat milk) at room temperature for 1 h. Then, the membranes were visualized using the enhanced chemiluminescence (ECL) reagent (Millipore, Billerica, MA, USA) and the chemiluminescence imaging system (ChemiScope 5300; Clinx Science Instruments, Shanghai, China). The band intensity was analyzed using a chemiluminescence imaging system (ChemiScope 5300; Clinx Science Instruments, Shanghai, China).

### 5.14. Statistical Analysis

Data were analyzed with SPSS 16.0 (SPSS Inc., Chicago, IL, USA) using one-way analysis of variance (ANOVA). Data were represented as mean ± SEM from triplicate independent experiments. A *p*-valve < 0.05 was considered to be significant.

## Figures and Tables

**Table 1:** Summary of bacterial strains, plasmids, and primers used in this study.

Strain or Plasmid	Characteristic or Function	Source
**Strains**		
Avian pathogenic *Escherichia coli* (APEC) XM	Virulent strain of APEC	Donated by Dr. Guoqiang Zhu, Yangzhou University
APEC XM Δ*clbG*	Deletion mutant of *clbG* with APEC XM background	This study
APEC XM Δ*clbG*/p*clbG*	APEC-XM Δ*clbG* with the vector pACYC184**-***clbG*, Cm^r^	This study
**Plasmid**		
pKD46	λ red recombinase expression plasmid	[90]
pKD3	pANTSγ derivative containing FRT-flanked, Cm^r^	[90]
pCP20	temperature-sensitive replication and thermal induction of FLP synthesis	[90]
pACYC184-*clbG*	pACYC184 containing the promoter followed by the full-length *clbG*, Cm^r^	This study
	Sequence (5′→3′)	Product size
P1	GTCCTCTTCGCTGGATGT	1150/275
P2	GAACATCAGTGCGACATC	
P3	TGCGCACTGGCAGCCACATCGGCGGCGCGGTGATGGCGTGTGGCTGTCTGTGTAGGCTGGAGCTGCTTC	1000
P4	GCTCCGGTTCGCAATATGTAGGCATGGCACGGTGGCTGTATGAGCGTTCATATGAATATCCTCCTTAG	
P5	CTAACGCAGTCAGGCACCGTGTATGACGAAGGATGTCGCACTGATG	1150
P6	GTGCCGCCGGCTTCCATTTACGCC TGTCCGCCGTTG	
*GAPDH*	AACGGGAAGCCCATCACCATC	98
	AAGACACCAGTAGACTCCACGA	
*IL-1β*	ATGAAAGACGGCACACCCAC	175
	GCTTGTGCTCTGCTTGTGAG	
*IL-6*	TGCAAGAGACTTCCATCCAGT	71
	GTGAAGTAGGGAAGGCCG	
*TNF-α*	ACTGAACTTCGGGGTGATCG	97
	TGATCTGAGTGTGAGGGTCTGG	

## Data Availability

The datasets generated and/or analyzed during the current study are not publicly available due to the project is not finished yet but are available from the corresponding author on reasonable request.
